# Activation of kappa opioid receptors reduces seizure activity in a dose-dependent way

**DOI:** 10.1186/2050-6511-13-S1-A91

**Published:** 2012-09-17

**Authors:** Luca Zangrandi, Christoph Schwarzer

**Affiliations:** 1 Department of Pharmacology, Innsbruck Medical University, 6020 Innsbruck, Austria

## Background

Neuropsychiatric disorders are one of the main challenges of human medicine with epilepsy as one of the most frequent. Temporal lobe epilepsy represents the most common type of epilepsies and is often accompanied by marked neuronal degeneration. One main factor that causes neural loss is the excitotoxicity of glutamate, which is copiously released during seizures and hypoxia accompanying seizures. It was also shown that the deletion of prodynorphin in mice and low expression in humans is associated with increased epilepsy vulnerability. Dynorphin targets opioid receptors and in particular the κ opioid receptor (KOP). The KOP receptors in the hippocampal formation are located in very strategically points for the control of glutamate release and, most importantly, they are not altered under epileptic conditions. Still, the functional background of these neuroprotective effects is not fully understood. The aim of this study was to investigate the influence of KOP agonists on EEG patterns of epileptic mice.

## Methods

Kainic acid (KA; 1 nmol in 50 nL saline) was injected unilaterally into the dorsal hippocampus, causing acute and delayed behavioral and EEG effects. Four-channel EEG traces were recorded from ipsi- and controlateral hippocampi and motor cortices applying depth and surface electrodes, respectively. The KOP-specific agonist U-50488H was dissolved in saline (adjusted to pH 7.4) and applied i.p.

## Results

Sharp waves and paroxysmal discharges in the ipsilateral hippocampus were recorded about 14 days after KA injection. Paroxysmal discharges were accompanied by behavioral arrest and stereotypic behavior like head nodding. Application of KOP agonists blocked paroxysmal discharges up to 2 hours in a dose-dependent manner, which was comparable to the effect of 2.5 mg/kg diazepam. Moreover, animals treated with U-50488H were awake.

## Conclusions

Data collected so far demonstrate the anticonvulsant action of KOP agonists in the chronic phase of epilepsy, suggesting that KOP agonists may represent potential drug targets for novel anti-epileptics.

